# Prediction of Morbidity and Mortality After Esophagectomy: A Systematic Review

**DOI:** 10.1245/s10434-024-14997-4

**Published:** 2024-02-21

**Authors:** M. P. van Nieuw Amerongen, H. J. de Grooth, G. L. Veerman, K. A. Ziesemer, M. I. van Berge Henegouwen, P. R. Tuinman

**Affiliations:** 1 Department of Adult Intensive Care Medicine, Amsterdam UMC (VUmc), Amsterdam, The Netherlands; 2 Medical Library, Vrije Universiteit, Amsterdam, The Netherlands; 3 Department of surgery, Amsterdam UMC, University of Amsterdam, Amsterdam, The Netherlands; 4 Cancer Center Amsterdam, Cancer Treatment and Quality of Life, Amsterdam, The Netherlands; 5 Amsterdam Institute for Immunology and Infectious Diseases, Amsterdam, The Netherlands

**Keywords:** Esophageal cancer, Esophagectomy, Prediction models, Mortality, Morbidity

## Abstract

**Background:**

Esophagectomy for esophageal cancer has a complication rate of up to 60%. Prediction models could be helpful to preoperatively estimate which patients are at increased risk of morbidity and mortality. The objective of this study was to determine the best prediction models for morbidity and mortality after esophagectomy and to identify commonalities among the models.

**Patients and Methods:**

A systematic review was performed in accordance to the Preferred Reporting Items for Systematic Reviews and Meta-Analyses statement and was prospectively registered in PROSPERO (https://www.crd.york.ac.uk/prospero/, study ID CRD42022350846). Pubmed, Embase, and Clarivate Analytics/Web of Science Core Collection were searched for studies published between 2010 and August 2022. The Prediction model Risk of Bias Assessment Tool was used to assess the risk of bias. Extracted data were tabulated and a narrative synthesis was performed.

**Results:**

Of the 15,011 articles identified, 22 studies were included using data from tens of thousands of patients. This systematic review included 33 different models, of which 18 models were newly developed. Many studies showed a high risk of bias. The prognostic accuracy of models differed between 0.51 and 0.85. For most models, variables are readily available. Two models for mortality and one model for pulmonary complications have the potential to be developed further.

**Conclusions:**

The availability of rigorous prediction models is limited. Several models are promising but need to be further developed. Some models provide information about risk factors for the development of complications. Performance status is a potential modifiable risk factor. None are ready for clinical implementation.

**Supplementary Information:**

The online version contains supplementary material available at 10.1245/s10434-024-14997-4.

Esophageal cancer is the sixth leading cause of cancer death worldwide.^1^ Treatment for patients with esophageal malignancies generally consists of neoadjuvant chemoradiation followed by esophagectomy, a high-risk procedure with a complication rate of up to 60%.^2–5^ These postoperative complications are associated with significant morbidity, mortality, and health economic effects. In addition to worse patient outcomes, healthcare costs for a complicated course can be 2.5 times higher than that for an uncomplicated course and 5% of patients are responsible for about 20% of total hospital costs.^6–11^

Early identification of patients at high risk of severe complications has three potentially important healthcare benefits. First, patients at high risk of complications or death can be better informed about potential adverse consequences of surgery, which may lead to alternative treatment strategies. Second, potential preventative measures can be tailored to the risk profile by influencing potentially modifiable risk factors. Third, patients at the highest risk levels can be monitored more closely (for example, using remote monitoring or high-care ward admission) for early detection and treatment of complications—interventions that might not be cost-effective for the whole population.

A large number of preoperative prediction models have been developed in recent years on morbidity and mortality after esophagectomy. These models could potentially be helpful in identifying high-risk patients, but their usefulness has not yet been assessed systematically. In many research areas, the number of developed prediction models far outpaces their implementation rate, and novelty often takes precedence over validity, robustness, and usefulness.^12–16^

The primary aim of this study was to evaluate which of the existing prediction models are most suitable for potential widespread implementation. To evaluate the potential usefulness and readiness for clinical practice, we integrated results of models’ predictive performance with methodological quality assessment and availability of the input variables.

The secondary aim was identification of commonalities among the best-performing models, in which the focus was on models predicting mortality and pulmonary complications.

## Patients and Methods

### Study Design

This is a systematic review. The conduct and reporting of this review adhere to the Preferred Reporting Items for Systematic Reviews and Meta-Analyses (PRISMA) statement (www.prisma-statement.org) and was prospectively registered in PROSPERO (https://www.crd.york.ac.uk/prospero/, study ID CRD42022350846).^17^

### Literature Search Strategy

Three bibliographic databases, PubMed, Embase.com, and Clarivate Analytics/Web of Science Core, were searched for relevant literature from inception to 25 August 2022. Searches were devised in collaboration with a medical information specialist (KAZ). Search terms, including synonyms, closely related words, and keywords, were used as index terms or free-text words: ‘esophagectomy’ and ‘prediction’. No methodological search filters, date, or language restrictions were applied that would limit results.

ASReview (version 1.0) was used to rank potentially relevant titles and abstracts. Screening in ASReview was carried out independently by two reviewers (MPvNA and GLV). All references marked as relevant were manually screened for eligibility by both reviewers. If necessary, the full-text article was checked for the eligibility criteria. Differences in judgement were resolved through a consensus procedure. If no consensus was reached, a third reviewer was consulted (PRT).

The full search strategy is detailed in Supplementary Material 1.

### Eligibility Criteria

Studies were included in which prognostic models/scales/indexes were developed and/or validated with respect to the preoperative prediction of morbidity (Clavien–Dindo score of at least 3) and/or mortality within 90 days after esophagectomy owing to esophageal cancer (regardless of histology or surgery type).^18^ All types of prediction modeling studies were included. We excluded articles written before 2010. To assess models that can be used today, it is desirable that the study population match the current patient population as much as possible. Models consisting of one type of variable (such as blood markers, nutritional status, or cardiopulmonary exercise testing) were excluded. Articles that only examined association and/or correlation between the score of a model and morbidity/mortality were excluded.

To compare the accuracy of models, only models that examined accuracy and reported an outcome measure, such as area under the receiver operating characteristic curve (AUC) or observed/expected ratio (O/E ratio), were included. For more details about the inclusion and exclusion criteria, see Supplementary Material 2. Outcome definitions were described in Supplementary Material 3.

### Assessment of Methodological Quality

Two reviewers (MPvNA and GLV) independently assessed methodological quality of full-text papers using the Prediction model Risk Of Bias ASsessment Tool (PROBAST).^19^ This tool, especially designed for systematic reviews of prediction models, assesses the risk of bias in four domains (participants, predictors, outcome, and statistical analysis) and addresses the concerns of applicability in three domains (participants, predictors, and outcome). A domain was assessed as low risk when all signaling questions were answered yes or probably yes. A domain was assessed as high risk when at least one signaling question in that domain was answered no or probably no. Overall risk of bias was assessed as low when all domains were considered low risk. Overall risk of bias was assessed as high when at least one domain was considered high risk. For domain one, participants, applicability is scored as unclear if it is unclear how many patients received neoadjuvant chemoradiation or if less than half the patients received neoadjuvant therapy.

When multiple models were developed and/or validated in a single study, a separate PROBAST form for each model or for both development and validation was needed. However, if results were completely similar, then this is reflected as one result in the Supplementary Material.

### Data Extraction

Data extraction of the identified studies was performed using the Critical Appraisal and Data Extraction for Systematic Reviews of Prediction Modelling Studies (CHARMS) checklist (MPvNA).^20^ Extracted data consisted of study characteristics (first author, country, study type, pretreatment, surgery type, and cohort years), study outcomes (outcome, number of events/sample size, outcome measures used regarding discrimination, and calibration), and the variables used in the different models.

### Data Synthesis and Statistical Analysis

The results were tabulated and a narrative synthesis was performed.

For prognostic accuracy (e.g., discrimination) the AUC value was often used. An AUC value under 0.60 was rated as poor, a value between 0.60 and 0.75 as possibly helpful discrimination, and more than 0.75 as useful discrimination.^21^ For calibration, the Hosmer–Lemeshow test was often used. Small *p*-values mean that the model has poor calibration.

To assess potential usefulness and readiness for clinical practice, we integrated the results of the methodological quality assessment with the predictive performance of the models (lower limit confidence interval AUC), presence of external validation, sample size, and availability of the input variables. We rated the models ranked high in the tables as the better models.

Commonalities of the models (predictor variables) were presented in a figure and quality assessments were transformed into figures using Rstudio, version 4.2.1.

## Results

### Systematic Search

**Fig. 1:**
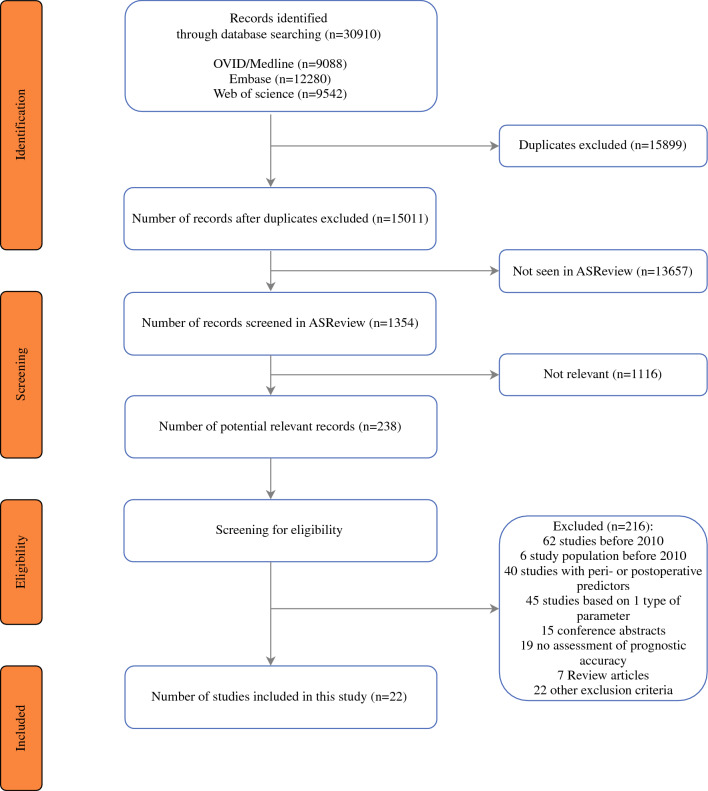
Flowchart of the study search and selection procedure

Details regarding the literature search are shown in Fig. 1. Of the original 15,011 references identified by the search, 22 articles met the inclusion criteria and were included in this systematic review, using data from 108,208 patients.

### Study Characteristics

Study characteristics are presented in Table 1. Seven studies were conducted in Asia, seven in Europe, six studies in the USA, and two studies were intercontinental studies. In general, data were retrospectively collected from an existing database. Of the 22 studies, 12 collected their data entirely from 2010 onward, and the remaining studies partly after 2010. Eleven studies included a population in which at least half of the patients were pretreated with chemotherapy, radiotherapy, chemoradiotherapy, or immunochemotherapy.

**Table 1:** Study characteristics

Study ID	Country/cohort years	Pretreatment (%)	Surgery type	Model: development and/or validation; sample size (*n*)	Validation of
*Mortality*					
D’Journo et al., 2021^36^	19 countries worldwide 2015–2019	CRTx: 47, CTx: 29, RTx: 0.1	MIE/open, TT/TH	Dev: 4172, Ext val: 4231	Dev model
Takeuchi et al., 2014^24^	Japan 2011	CTx: 19, RTx: 5	total/hybrid MIE/OE, TT/TH, partial unknown	Dev: 4261, Ext val: 1093	Dev models
Sasaki et al., 2023^25^	Japan 2012–2017	RTx: 7, CTx: Unclear	TT	Dev: 29501, Ext val: 3278	Dev models
Fischer et al., 2016^51^	UK 2011–2013	?	?	Dev: 4882, Int val: 800 bootstrapsamples	Dev models
Fuchs et al., 2017^52^	USA 1998–2011	?	MIE/open, TT/TH	Dev: 23751	NA
D’Journo et al., 2017^23^	France 2004–2013	CTx: 17, RTx: 0.4, CRTx: 23	TH/TT/TA	Val: 1039	Steyerberg
Raymond et al., 2016^53^	USA 2012–2014	CTx or RTx: 68	MIE/open, TT/TH/TA	Rev STS GTSD model: 3942	NA
Wan et al., 2022^44^	USA 2006–2017	?	?	Val: 10602	RAI-rev, RAI-A, mFI-5, Rev of RAI-rev (cancer corrected)
Fodor et al., 2015^29^	Romania 2011–2014	?	?	Val: 55	O-POSSUM, ASA
*Morbidity or both morbidity and mortality*					
Filip et al., 2015^27^	Italy 2008–2012	CRTx: 78	MIE, TT/TH	Dev new model and val existing models: 167/500 bootstrapsamples	aCCI, CCI, O-POSSUM, ASA, Lagarde, new developed Padua model
Raymond et al., 2016^53^	USA 2012–2014	CTx or RTx: 68	MIE/open, TT/TH/TA	Rev STS GTSD model: 3942	NA
Wan et al., 2022^44^	USA 2006–2017	?	?	Val: 10602	RAI-rev, RAI-A, mFI-5, Rev of RAI-rev (cancer corrected)
Saito et al., 2019^26^	Japan 2007–2015	CTx: 32	MIE, TT	Dev: 90	NA
Scarpa et al., 2016^30^	Italy 2008–2012	CRTx: 79	MIE, TT	Val: 181	aCCI, CCI, ASA
Filip et al., 2014^28^	Romania 2004–2013	CRTx: 51	TH/TT	Val: 43	aCCI, CCI, O-POSSUM
Mora et al., 2021^31^	Japan 2010–2016	CTx: 14, CRTx: 5	MIE/open, TT (3FLD, McKeown)	Val: 230	aCCI, CCI, O-POSSUM, Steyerberg
Gray et al., 2023^54^	USA 2016–2018	?	MIE, TT	Val: 240	ACS NSQIP calculator, mFI-5
Ravindran et al., 2020^55^	USA 2013–2017	Neoadjuvant: 90	TT	Val: 100	ACS NSQIP calculator
*Anastomotic leakage*					
Fischer et al., 2016^51^	UK 2011–2013	?	?	Dev: 4882, Int val: 800 bootstrapsamples	Dev models
Van Kooten et al., 2022^35^	The Netherlands 2011–2017	CTx: 7%, CRTx: 86%	TT/TH	Dev: 3171, Ext val: 1057	Dev models
Ohkura et al., 2019^22^	Japan 2011–2012	?	?	Dev: 8715, Ext val: 2147	Dev models
*Pulmonary complications*					
Thomas et al., 2019^33^	Belgium & USA 2002–2017	CRTx: 100	MIE/open, TT	Dev: 601, Ext val: 90	Dev model
Van Kooten et al., 2022^35^	The Netherlands 2011–2017	CTx: 7%, CRTx: 86%	TT/TH	Dev: 3171, Ext val: 1057	Dev models
Ohkura et al., 2019^22^	Japan 2011–2012	?	?	Dev: 8715, Ext val: 2147	Dev models
Kanda et al., 2019^56^	Japan 2005–2017	CTx: 52	MIE/open	Dev: 355	NA
Wang et al., 2022^32^	China 2019–2021	ICTx: 100	MIE/open, TT	Dev: 78	NA
Reinersman et al., 2016^34^	USA 2009–2012	CRTx: 80	Total/hybrid MIE, TH/TT	Val: 136	Ferguson

*Dev* development, *Val* validation, *Int* internal, *Ext* external, *NA* not applicable, *CTx* chemotherapy, *CRTx* chemoradiotherapy, *ICTx* immunochemotherapy, *MIE* minimal invasive esophagectomy, *OE* open esophagectomy, *TT* transthoracal, *TH* transhiatal, *TA* thoracoabdominal, *RAI* risk analysis index, *Rev* revised, *RAI-A* administrative risk analysis index, *mFI-5* 5-factor modified frailty index, *STS GTSD* Society of Thoracic Surgeons General Thoracic Surgeons Database, *aCCI* age adjusted Charlson comorbidity index, *CCI* Charlson Comorbidity Index, *ASA* American Society of Anesthesiologists, *O-POSSUM* physiological and operative severity score for the enumeration of mortality and morbidity adjusted for oesophagogastric surgery, *ACS NSQIP* American College of Surgeons National Surgical Quality Improvement Program, *?* unknown

Most articles described the development of one or more new models or described the validation of one or more existing models. We assessed 39 models, including 8 models in the study by Ohkura et al.^22^ From this study, only the two most relevant models were used in our analysis (anastomotic leakage and pneumonia). Finally, 33 models were included in this systematic review, of which 18 models were newly developed.

### Quality Assessment

The overall risk of bias (ROB) and overall concerns regarding applicability are presented in Table 2. For more details about ROB and concerns regarding applicability, see Supplementary Material 4.

**Table 2:**
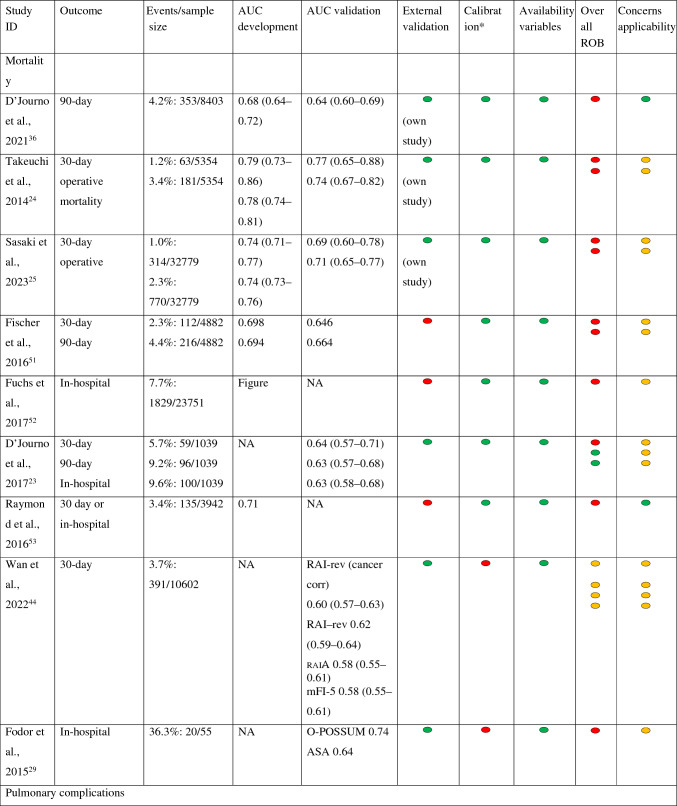

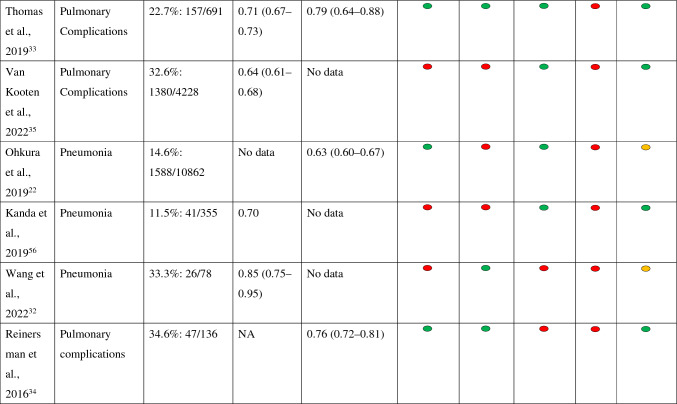
Summary of evaluation of prediction models for mortality and pulmonary complications

*The studies by Takeuchi et al. and Raymond et al. described that calibration had been done, but no data were shown

*NA* not available

Risk of bias:

- Participants: For the development and validation of the models, retrospective cohort and/or existing (national) databases were generally used. Most studies showed a low risk of bias at this point.
- Predictors: There does not appear to be a risk of bias in any of the studies.
- Outcome: A few studies had unclear risk of bias owing to unclear definitions of outcome.
- Analysis: Most studies had a high risk of bias, in developmental studies mainly owing to an insufficient event-to-variable rate (candidate variable), and in validation studies mainly owing to an insufficient event rate (less than 100 events). Development studies on mortality have been carried out in large populations. This is in contrast to some development studies related to pulmonary complications. With the exception of the validation study by D'Journo et al., the studies validating only preexisting models were carried out in small populations.^23^
  Other common causes of risk of bias in development studies were lack of relevant model performance measures or lack of correction for overfitting or optimism.
  A model may be overfit when it makes good predictions on the study sample (owing to certain typical factors in the study population) but poor predictions outside of the study sample. This can be corrected through techniques such as bootstrapping or cross-validation.

Concerns regarding applicability: Concerns regarding applicability were in general related to no information whether neoadjuvant chemoradiation was given or because there were relatively few people in the population that underwent neoadjuvant treatment with chemoradiation.

### Discrimination, Calibration, and Validation

Study outcomes are presented in Table 2. More detailed information can be found in Supplementary Material 5.

#### Discrimination

Discrimination of models for mortality: Only Takeuchi’s models had a prognostic accuracy of about or greater than 0.75; Sasaki’s models were just below that.^24,25^ The remaining models had accuracies between 0.60 and 0.75.

Discrimination of models for morbidity and/or mortality: Only Saito’s model, the Padua model, the aCCI validated by Filip, and the O-POSSUM model validated by Fodor found prognostic accuracies between 0.74 and 0.80.^26–29^ Other models had poor performance, including the aCCI validated in studies other than Filip’s.^27,30,31^

Discrimination of models for pulmonary complications: The development of Wang’s model, the validation of Thomas’ model, and the Ferguson model found accuracies above 0.75. All were done using small sample sizes.^32–34^ The remaining models found accuracies between 0.60 and 0.75.

Discrimination of models for anastomotic leakage: All three newly developed models found accuracies between 0.53 and 0.63.

#### Calibration

Calibration was reported most as a non-significant Hosmer–Lemeshow test^6^ or a figure such as a scatterplot or calibration plot.^5^ Eight studies did not report on calibration and one study indicated a favorable correlation between predicted and observed events, but the data were not shown.^24^ For more details about calibration, see Supplementary Material 5.

#### Validation

None of the 18 newly developed models were validated by another research group, 14 models were validated by the author’s own research group (in a new population or by bootstrapping), and 4 models were developed but not validated. Additionally, 11 existing models were validated one or more times. For more details, see Supplementary Material 6.

### Predictor Variables

**Fig. 2:**
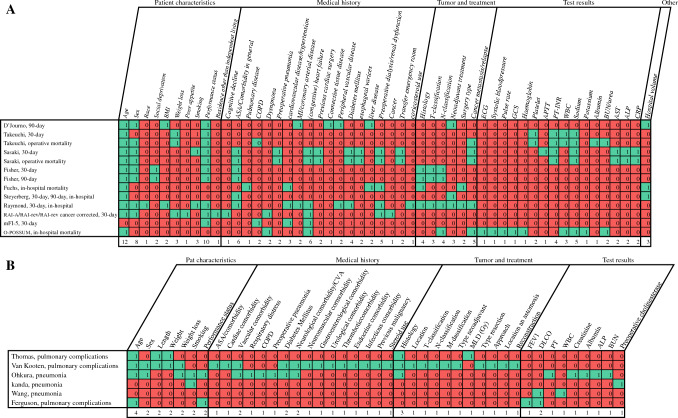
**A** Mortality predictor variables; **B** predictor variables of pulmonary complications

An overview of the different predictor variables is presented in Fig. 2A,B.

For the prediction models on mortality, 52 different variables were used with a median of 10 variables per model (range 4–20). Most studies used eight or more predictor variables. The easiest model to use is Steyerberg’s model with just four easily available predictor variables.^23^ The predictor variables could be classified as patient characteristics, medical history, tumor and treatment, test results, or other. Age, sex, and performance status were the predictor variables most used regarding patient characteristics. ASA/comorbidity in general, (congestive) heart failure, and preoperative dialysis/renal dysfunction were the predictor variables most used regarding medical history. Histology, N-classification, and cancer metastasis/relapse were most used regarding tumor and treatment. PT-INR, white blood cell count, and sodium were most used regarding test results. Hospital volume was included in three models.

For the pulmonary complication prediction models, 43 different variables were used with a median of 5 predictors per model (range 2–28). Most models used less than five predictors. Exceptions were Van Kooten’s model regarding pulmonary complications which had 28 predictor variables, and Ohkura’s model, which had 17 predictor variables.^22,35^ Again, variables could be classified as patient characteristics, medical history, tumor and treatment, or test results. Only age and histology were used in more than two models.

For most models, variables are easily available, making the models relatively easy to use (Table 2). Only the models of Ferguson (studied by Reinersman et al.) and Wang require a pulmonary function test, which is not performed routinely in all patients.^32,34^

### Assessment of the Best Models

On the basis of these results, we could conclude that there are a number of models that have the potential to be developed further. For models that predict mortality, the most promising models are the models by D'Journo et al. and Takeuchi et al.^24,36^ On the basis of quality assessment, there is a risk of bias, but by weighing this against the other points of assessment (validation of a model in a sample separate from the development cohort, height of lower limit 95% confidence interval AUC, generalizability of the study, and sample size), these seem to be the better models. On the basis of the results, for models predicting pulmonary complications, the model by Thomas et al. is the best performing model.^33^ For anastomotic leakage, a model with potential has yet to be developed (partly owing to the fact that all three models had an AUC lower than 0.64).

## Discussion

The major findings of this systematic review assessing prediction models for complications after esophagectomy are that there are several models that are either promising to be further developed or provide us with the information about risk factors for the development of complications. Models with the most potential regarding prediction of mortality are the models by D’Journo and Takeuchi, while Thomas’s model has the most potential regarding pulmonary complications. However, none of these three models have been validated by independent investigators yet.

Although it may be too early to implement complication prediction models in clinical practice, given the often relatively low AUC, the risk of bias, etc., the mortality models do at least provide us with relevant information about variables that influence the mortality risk. Common predictor variables in mortality models include age, sex, performance status, ASA score/comorbidity in general, and cancer metastasis/relapse. Of these factors, possibly only performance status could be influenced prior to surgery. This could mean two things: either a poor performance status could be examined preoperatively to see whether it could be improved before the esophagectomy, or performance status, if it is not yet, could be given a role, as with the non-influenceable factors age, sex, comorbidity, and cancer metastasis/relapse, in the preoperative assessment as to whether a surgery is the best option for the patient. In conversation with the patient, however, we should remain cautious regarding statements about the severity of the risk of complications. While we do know that a number of single factors can affect risk, we do not know what the exact level of risk is when multiple risk factors are present.

It is notable that in models for pulmonary complications, there is no uniformity in which variables should be included in models. More research is needed for this.

When examining model variables, it is noteworthy that not all models incorporate factors known to be linked with mortality and morbidity, such as surgical technique and hospital volume.^2,37–39^ A possible explanation is that surgical technique is not regarded as a preoperative variable, and hospital volume is not considered a patient-specific variable. Additionally, if the entire population comprises patients treated with the same technique, it is logical that the technique may not be included as a variable. Therefore, we recommend considering surgical technique as a potential variable in models for populations with diverse techniques when developing or revising a model.

A recently published systematic review also focused on preoperative prediction models for complications after esophagectomy.^40^ While that study included all studies from 2000, our study focused on populations as close as possible to current patient populations in terms of neoadjuvant treatment, etc. We used a more robust quality assessment tool and provided a more detailed description of quality assessment.^19,41^ As a result, we rated more studies as high risk of bias.

Generalizability issues are a major risk in all assessed prediction studies, a problem that is inherent to the topic. We included a large number of prediction models and data of more than 100,000 patients. However, the low event rate of post-esophagectomy mortality (usually below 5%, and in large centers below 1%) substantially decreases the effective sample size available for risk factor identification and prediction modelling in each individual study. None of the validation studies reported a sample size calculation or used the simple rule of thumb of at least 100 events in the study population.^42,43^ Only the validation studies performed by D’Journo et al. and Wan et al. met the aforementioned rule of thumb.^23,44^ Additionally, very lengthy study periods to obtain a workable sample size (up to 15 years in some studies) can mean that some data are outdated by the time of publication, as diagnostics, operative techniques, and postoperative treatment protocols have changed.

None of the 18 developed models have been validated outside their own research group. This is a more widely known problem in prediction modeling, as only 15% of developed prediction models are externally validated.^45^ AUC’s in external validation studies are generally lower than in the development study and never increase by more than 0.03. This means that the real-world accuracy could not be assessed for any of the models.

Eight studies did not report on model calibration and one study indicated a favorable correlation between predicted and observed events.^24^ Calibration indicates the extent to which the predicted proportions of the event match the actually observed proportions of the event and is particularly important when a model will be used to support a decision.^16^

### Strengths and Limitations

This study has several strengths. To our knowledge, this systematic review is the most recent and thorough systematic review on preoperative morbidity and mortality prediction models to date. Moreover, we registered our study at PROSPERO in advance. Previous systematic reviews were either written several years ago, cover prognosis of esophageal cancer in general, describe only mortality as outcome, are about long-term survival, or include models with perioperative rather than preoperative variables.^46–50^

One of the limitations of this study is that the pretreatment was not clearly stated, or the pretreatment turned out to be radiation, chemotherapy or immunochemotherapy, or chemoradiation was given in just a small proportion of patients.

In conclusion, the availability of rigorous prediction models is limited and none are ready for clinical implementation. Several models are promising but need to be further developed. In addition, some models provide us with the information regarding risk factors for the development of complications. Performance status is a potential modifiable risk factor when it comes to reducing risk of morbidity and mortality.

### Supplementary Information

Below is the link to the electronic supplementary material.

Supplementary file 1
