# Supplementary material for: Prediction of Morbidity and Mortality After Esophagectomy: A Systematic Review
Source: Ann Surg Oncol. 2024 Feb 21;31(5):3459–70. doi: 10.1245/s10434-024-14997-4 (PMC10997705; doi:10.1245/s10434-024-14997-4)
Supplement: Supplementary file 1 [file 10434_2024_14997_MOESM1_ESM.docx]

**Supplemental material 1 Search strategy**

**OVID/Medline Results (25 August 2022)**

| **Search** | **OVID/Medline Query – August 25, 2022** | **Results** |
| --- | --- | --- |
| 1 | exp esophagectomy/ or ((esophag* or oesophag*) adj3 (surger* or anastom* or resect* or reconstruct* or transect* or operat*)).ti,ab,kf. or (esophagect* or oesophagect*).ti,ab,kf. | 27552 |
| 2 | (model* or regression* or network* or predict* or prognos* or risk or nomogram*).ti,ab,kf. | 7664568 |
| 3 | 1 and 2 | 9088 |

**Embase.com Results (25 August 2022)**

| **Search** | **Embase Query – August 25, 2022** | **Results** |
| --- | --- | --- |
| #5 | #4 NOT 'conference abstract'/it | 12280 |
| #4 | (#1 OR #2) AND #3 | 16832 |
| #3 | model*:ti,ab,kw OR regression*:ti,ab,kw OR network*:ti,ab,kw OR predict*:ti,ab,kw OR prognos*:ti,ab,kw OR risk:ti,ab,kw OR nomogram*:ti,ab,kw | 10147320 |
| #2 | esophagect*:ti,ab,kw OR oesophagect*:ti,ab,kw | 20232 |
| #1 | 'esophagus resection'/exp OR 'esophagus surgery'/exp OR (((esophag* OR oesophag*) NEAR/3 (surger* OR anastom* OR resect* OR reconstruct* OR transect* OR operat*)):ti,ab,kw) | 53678 |

**Clarivate Analytics/Web of Science Core Collection Results (25 August 2022)**

| **Search** | **Web of Science Query – August 25, 2022** | **Results** |
| --- | --- | --- |
| #3 | #1 AND #2 | 9542 |
| #2 | TS=(model* OR regression* OR network* OR predict* OR prognos* OR risk OR nomogram*) | 15884163 |
| #1 | TS=((esophag* OR oesophag*) NEAR/3 (surger* OR anastom* OR resect* OR reconstruct* OR transect* OR operat*)) OR TS=(esophagect* OR oesophagect*) | 24654 |

Duplicate articles were excluded using the R-package ‘ASYSD’ an automated deduplication tool followed by manual deduplication in Endnote (X20.0.3) by the medical information specialist (KAZ). (54)

ASReview (version 1.0) was used to apply a ranking to the potentially relevant titles and abstracts using the default settings (Feature extraction technique: TF-IDF, Classifier: Naïve Bayes, Query Strategy: maximum, Balance Strategy: dynamic resampling (double) of an active learning algorithm.(55) ASReview was used to ensure that the search strategy could remain as broad as possible (that is, only search on synonyms of ‘esophagectomy’ and ‘prediction model’ and not, for example, on type of complications, as certain complications could then be missed).

**Supplemental material 2 In- and exclusion criteria**

Retrospective and prospective studies regarding development and internal or external validation of prediction models were included. Only original research article in a peer-reviewed journal were eligible for this review. Secondary research, reviews, conference proceedings, dissertations, editorials, expert opinions or consensus paper abstracts were excluded. All types of prediction modelling studies were included: Prediction model development studies without external validation in independent data, prediction model development studies with external validation in independent data and external model validation studies.

Studies were included in which models/scales/indexes have been developed and/or validated with respect to the preoperative prediction of mortality and/or morbidity after esophagectomy due to esophageal cancer (regardless of surgery type). Models that include perioperative and/or postoperative variables were excluded.

Both models that provide as an outcome an individual probability of morbidity/mortality for a patient and models where a number on a scale is obtained were included.

Regarding severity of complications: Only models were included that have as an outcome complications with a severity comparable to a Clavien Dindo score of at least 3 or higher.(18)

Regarding mortality: Only models were included in which the outcome is mortality within 90 days of surgery. Models involving mortality/survival after 90 days were excluded.

When models are developed for a broader group of patients than just patients after esophagectomy, only those models were included in which at least half the population consisted of patients after esophagectomy, or when the outcome measures were extractable specifically for esophagectomy.

Only models consisting of two or more different types of variables were included. Models that include only biomarkers as variables or that look only at nutritional status or cardiopulmonary exercise testing were therefore excluded.

Given that since about 2010 the treatment prior to esophagectomy is chemoradiation,

only articles written after 2010 describing a population that potentially at least partially consisted of patients included from 2010 onwards were included.(56)

To compare the accuracy of models, only models that actually examined accuracy and described an outcome measure such as Area under the receiver operating characteristic curve (AUROC) or observed/expected ratio (O/E ratio) were included. Articles that only examined association and/or correlation between the outcome on a model and morbidity/mortality were excluded.

Common serious complications are pneumonia and anastomotic leakage. If multiple models were developed in 1 study that each predict a separate complication, we look only at models that predict anastomotic leakage and pneumonia. If multiple models are validated in a validation study and some models are outside the inclusion criteria then only the data from the models that were within the inclusion criteria were extracted from this study.

**Supplemental material 3 Outcome definitions**

**3.1 Mortality**

Different mortality outcomes were studied: 30-day, 90-day, operative mortality or in-hospital mortality. Operative mortality was defined as death within the period of hospitalization or within 90 days (Sasaki) or death within the index hospitalization period up to 90 days or death after discharge within 30 days (Takeuchi). (24, 25)

**3.2 Morbidity**

The morbidity prediction models were models with a several probable outcomes. Sometimes a list of complications was given where the outcome of the model was the probability of one of those complications. In other cases, the outcome of the model was the probability of a complication with a certain Clavien Dindo score, where a Clavien Dindo score of three or higher was referred to as a serious complication and a score of 5 is "dead" (varies by model whether a score of 5 is included).(18)

**3.3 Anastomotic leakage**

Anastomotic leakage was defined as any clinically or radiologically proven disruption to the anastomosis.

**3.4 Pulmonary complications**

In three models, the outcome was specifically pneumonia; the other three models involved pulmonary complications in general.

**Supplemental material 4 Risk of bias and Concerns applicability**


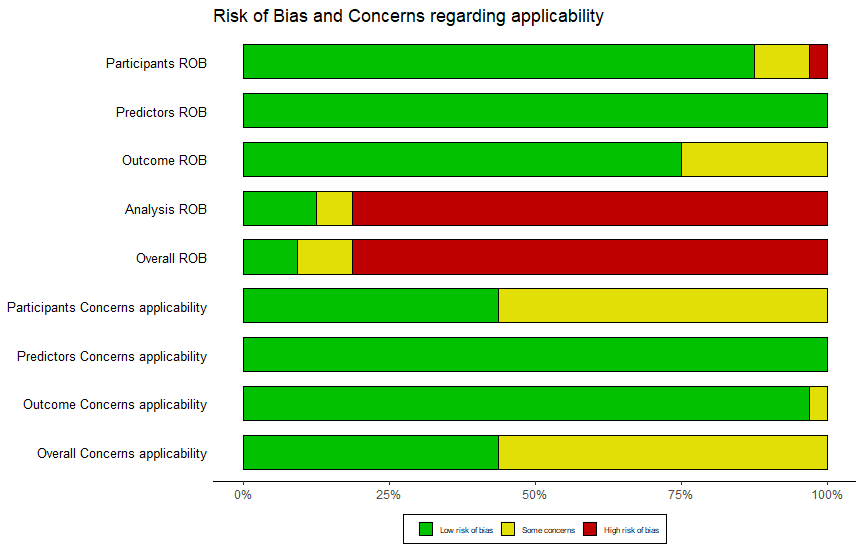


Risk of Bias and Concerns applicability


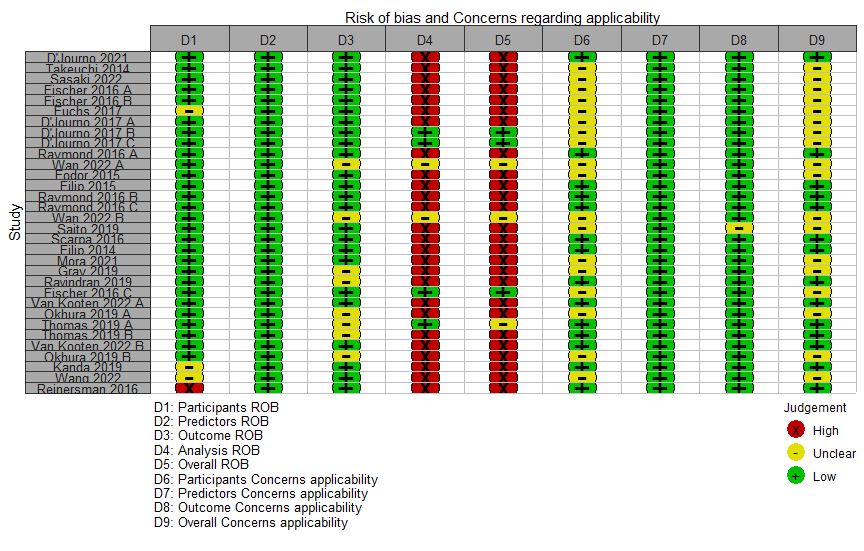


Legend: Fischer A: 30-day mortality, Fischer B: 90-day mortality, Fischer C: Anastomotic leakage, D’Journo A: 30-day mortality, D’Journo B: 90-day mortality, D’Journo C: in-hospital mortality, Raymond A: Mortality, Raymond B: Morbidity, Raymond C: Morbidity/mortality, Wan A: 30-day mortality, Wan B: morbidity, Van Kooten A: Anastomotic leakage, Van Kooten B: Pulmonary complications, Ohkura A: Anastomotic leakage, Ohkura B: Pneumonia, Thomas A: Model development, Thomas B: Model validation

Risk of bias:

- Participants: For the development and validation of the models generally retrospective cohort and/or existing (national) databases were used. Most studies showed a low risk of bias, sometimes an unclear risk due to unclear reasons as to whether and why patients were excluded and one study had a high risk due to exclusion of more than half of all patients without data on pulmonary function tests (which was necessary to calculate the risk of pulmonary complications).
- Predictors: There does not appear to be a risk of bias in any of the studies. Because models are often developed in large (pre-existing) databases, the variables are fairly general and unambiguous.
- Outcome: A few studies had unclear risk of bias due to unclear definitions of outcome.
- Analysis: Most studies had a high risk of bias, in developmental studies mainly due to an insufficient event-to-variable rate (candidate variable), in validation studies mainly due to an insufficient event rate (less than 100 events). Development studies on mortality have been done in large populations. This is in contrast to some development studies related to pulmonary complications. There, the sample size is so small that the studies have insufficient statistical power. With the exception of the validation study by d'Journo et al, the studies validating only pre-existing models have been done in small populations.(23)
- Other common causes of risk of bias in development studies were lack of relevant model performance measures, or lack of correction of overfitting or optimism.

Concerns regarding applicability: When there were some concerns regarding applicability it was generally related to no information whether neoadjuvant chemoradiation was given or there were relatively few people in the population that underwent neoadjuvant treatment with chemoradiation.

**Supplemental material 5 Study characteristics and outcomes**

**Study characteristics**

| **Study ID** | **Country** | **Model: development and/or validation:**  **Sample sizen (n)** | **Validation of** | **Pretreatment (%)** | **Surgery type** | **Cohort years** |
| --- | --- | --- | --- | --- | --- | --- |
| ***Mortality*** | | | | | | |
| **D'Journo et al, 2021 (36)** | 19 countries worldwide | Dev: 4172,  Ext val: 4231 | Dev model | CRTx: 47, CTx: 29, RTx: 0.1 | MIE/open, TT/TH | 2015-2019 |
| **Takeuchi et al, 2014 (24)** | Japan | Dev: 4261,  Ext val: 1093 | Dev models | CTx: 19, RTx: 5 | total/hybrid MIE/OE, TT/TH, partial unknown | 2011 |
| **Sasaki et al, 2023 (25)** | Japan | Dev: 29501,  Ext val: 3278 | Dev models | RTx: 7, CTx: Unclear | TT | 2012-2017 |
| **Fischer et al, 2016 (48)** | UK | Dev: 4882,  Int val: 800 bootstrapsamples | Dev models | ? | ? | 2011-2013 |
| **Fuchs et al, 2017 (49)** | USA | Dev: 23751 | N.a. | ? | MIE/open, TT/TH | 1998-2011 |
| **D'Journo et al, 2017 (23)** | France | Val: 1039 | Steyerberg | CTx: 17, RTx: 0.4, CRTx: 23 | TH/TT/TA | 2004-2013 |
| **Raymond et al, 2016 (50)** | USA | Rev STS GTSD model: 3942 | N.a. | CTx or RTx: 68 | MIE/open, TT/TH/TA | 2012-2014 |
| **Wan et al, 2022 (41)** | USA | Val: 10602 | RAI-rev, RAI-A, mFI-5, Rev of RAI-rev (cancer corrected) | ? | ? | 2006-2017 |
| **Fodor et al, 2015 (29)** | Romania | Val: 55 | O-POSSUM, ASA | ? | ? | 2011-2014 |
| ***Morbidity or both morbidity and mortality*** | | | | | | |
| **Filip et al, 2015 (27)** | Italy | Dev new model and val existing models: 167/500 bootstrapsamples | aCCI, CCI, O-POSSUM, ASA, Lagarde, new developed Padua model | CRTx: 78 | MIE, TT/TH | 2008-2012 |
| **Raymond et al, 2016 (50)** | USA | Rev STS GTSD model: 3942 | N.a. | CTx or RTx: 68 | MIE/open, TT/TH/TA | 2012-2014 |
| **Wan et al, 2022 (41)** | USA | Val: 10602 | RAI-rev, RAI-A, mFI-5, Rev of RAI-rev (cancer corrected) | ? | ? | 2006-2017 |
| **Saito et al, 2019 (26)** | Japan | Dev: 90 | N.a. | CTx: 32 | MIE, TT | 2007-2015 |
| **Scarpa et al, 2016 (30)** | Italy | Val: 181 | aCCI, CCI, ASA | CRTx: 79 | MIE, TT | 2008-2012 |
| **Filip et al, 2014 (28)** | Romania | Val: 43 | aCCI, CCI, O-POSSUM | CRTx: 51 | TH/TT | 2004-2013 |
| **Mora et al, 2021 (31)** | Japan | Val: 230 | aCCI, CCI, O-POSSUM, Steyerberg | CTx: 14, CRTx: 5 | MIE/open, TT (3FLD, McKeown) | 2010-2016 |
| **Gray et al, 2023 (51)** | USA | Val: 240 | ACS NSQIP, mFI-5 | ? | MIE, TT | 2016-2018 |
| **Ravindran et al, 2020 (52)** | USA | Val: 100 | ACS NSQIP calculator | Neoadjuvant: 90 | TT | 2013-2017 |
| ***Anastomotic leakage*** | | | | | | |
| **Fischer et al, 2016 (48)** | UK | Dev: 4882,  Int val: 800 bootstrapsamples | Dev models | ? | ? | 2011-2013 |
| **Van Kooten et al, 2022 (35)** | The Netherlands | Dev: 3171,  Ext val: 1057 | Dev models | CTx: 7%, CRTx: 86% | TT/TH | 2011-2017 |
| **Ohkura et al, 2019 (22)** | Japan | Dev: 8715, Ext val: 2147 | Dev models | ? | ? | 2011-2012 |
| ***Pulmonary complications*** | | | | | | |
| **Thomas et al, 2019 (33)** | Belgie & USA | Dev: 601,  Ext val: 90 | Dev model | CRTx: 100 | MIE/open, TT | 2002-2017 |
| **Van Kooten et al, 2022 (35)** | The Netherlands | Dev: 3171,  Ext val: 1057 | Dev models | CTx: 7%, CRTx: 86% | TT/TH | 2011-2017 |
| **Ohkura et al, 2019 (22)** | Japan | Dev: 8715, Ext val: 2147 | Dev models | ? | ? | 2011-2012 |
| **Kanda et al, 2019 (53)** | Japan | Dev: 355 | N.a. | CTx: 52 | MIE/open | 2005-2017 |
| **Wang et al, 2022 (32)** | China | Dev: 78 | N.a. | ICTx: 100 | MIE/open, TT | 2019-2021 |
| **Reinersman et al, 2016 (34)** | USA | Val: 136 | Ferguson | CRTx: 80 | Total/hybrid MIE, TH/TT | 2009-2012 |

Table legend: Dev: Development, Val: Validation, Int: Internal, Ext: External, N.a.: Not applicable, CTx: Chemotherapy, CRTx: Chemoradiotherapy, ICTx: Immunochemotherapy, MIE: Minimal Invasive Esophagectomy, OE: Open esophagectomy TT: Transthoracal, TH: Transhiatal, TA: Thoracoabdominal, RAI: Risk Analysis Index, Rev: Revised, RAI-A: administrative Risk Analysis Index, mFI-5: 5-factor modified Frailty Index, STS GTSD: Society of Thoracic Surgeons General Thoracic Surgeons Database, aCCI: Age adjusted Charlson Comorbidity Index, CCI: Charlson Comorbidity Index, ASA: American Society of Anesthesiologists, O-POSSUM: Physiological and Operative Severity Score for the enumeration of Mortality and morbidity adjusted for oesophagogastric surgery, ACS NSQIP: American College of Surgeons National Surgical Quality Improvement Program, ?: Unknown.

**Study outcomes**

| **Study ID** | **Outcome** | **Number events/total sample size** | **Number events split dev/val** | **Duration follow-up** | **Outcome measures discrimination** | **Model discrimination development** | **Model discrimination validation** | **Outcome measures calibration** | **Model**  **calibration development** | **Model cal validation** | **Model presentation** |
| --- | --- | --- | --- | --- | --- | --- | --- | --- | --- | --- | --- |
| ***Mortality*** | | | | | | | | | | | |
| **D'Journo et al, 2021 (36)** | 1 model: 90-day mortality | 353/8403 | 184/169 | 90 days | AUC | 0.68 (0.64-0.72) | 0.64 (0.60-0.69) | HL-test | not significant | not significant | Equation |
| **Takeuchi et al, 2014 (24)** | 2 models: 30-day and operative mortality | 30-day: 63/5354, operative: 181/5354. | N.a. | Until 30-day or until 90-day in case of prolonged hospital stay | AUC | 30-day: 0.79 (0.73–0.86), operative: 0.78 (0.74–0.81) | 30-day: 0.77 (0.65–0.88) operative: 0.74 (0.67-0.82) | No data | No data | No data | Equations |
| **Sasaki et al, 2023 (25)** | 2 models: 30-day mortality, operative mortality | 30-day: 314/32779, operative: 770/32779 | N.a. | 90 days or hospital stay | AUC | 30-day mortality: 0.74 (0.71–0.77), operative mortality: 0.74 (0.73–0.76) | 30-day mortality: 0.69 (0.60–0.78), operative mortality: 0.71 (0.65–0.77) | Calibration plot | Calibration plot | Calibration plot | Equation |
| **Fischer et al, 2016 (48)** | 3 models: 30-day mortality, 90-day mortality, anastomotic leakage | 30-day: 112/4882,  90-day: 216/4882, AL: 305/4882 | N.a. | mortality: 90-day, anastomotic leakage: Unknown | AUC | 30-day: 0.70, 90-day: 0.69, AL: 0.63 | 30-day: 0.65, 90-day: 0.66, AL: 0.59 | Scatterplot | No data | No data | Equations |
| **Fuchs et al, 2017 (49)** | 1 model: In-hospital mortality | 1829/23751 | N.a. | Hospital stay | sens/spec/  Youden index | Figure | No validation | Figure/table observed/expected | Figure | No validation | Risk scale |
| **D'Journo et al, 2017 (23)** | 1 model: 30-day, 90-day and in-hospital mortality | 30-day: 59/1039,  90-day: 96/1039,  IHM: 100/1039 | N.a. | 90 days | AUC | N.a. | 30-day: 0.64 (0.57–0.71), 90-day: 0.63 (0.57–0.68), IHM: 0.63 (0.58–0.68). | HL-test | N.a. | 30-day: HL significant in 4 deciles of patients, 90-day and IHM: Not significant | Equation |
| **Raymond et al, 2016 (50)** | 3 models: 30-day/IHM, morbidity, mortality and/or morbidity | mortality: 135/3942, morbidity: 1429/3942 | N.a. | Mortality: 30-day/IH, morbidity: n.a. | AUC | mortality: 0.71, morbidity: 0.63, mortality and/or morbidity: 0.63 | No validation | HL-test | No data | No data | Equations |
| **Wan et al, 2022 (41)** | 4 models: 30-day morbidity and 30-day mortality | Mortality: 391/10602, morbidity: 4234/10602 | N.a. | 30 days | AUC | N.a. | Morbidity: RAI-rev (cancer corrected) 0.51 (0.50-0.53), RAI-rev 0.54 (0.53-0.56), RAI-A 0.54 (0.53-0.55), mFI-5 0.57 (0.56-0.58). Mortality: RAI-rev (cancer corrected) 0.60 (0.57-0.63), RAI-rev 0.62 (0.59-0.64), RAI-A 0.58 (0.55-0.61), mFI-5 0.58 (0.55-0.61) | No data | N.a. | No data | Risk scales |
| **Fodor et al, 2015 (29)** | 2 models: Mortality during postoperative hospital stay | Mortality: 20/55 | N.a. | Hospital stay | AUC, sens/spec | N.a. | O-POSSUM: AUC 0.74, Sens 70%, Spec 71.4%, ASA: AUC 0.64, Sens 80%, Spec 51.4% | No data | N.a. | No data | O-POSSUM: Equation, ASA: Risk scale |
| ***Mortality and morbidity*** | | | | | | | | | | | |
| **Filip et al, 2015 (27)** | 1 model: 90-day morbidity, 5 models: minor complications, major complications | Minor complications: 39/167, major complications: 20/167, mortality: 2/167 | N.a. | 90 day | AUC | 90-day morbidity: 0.80 | aCCI: 0.61, CCI: 0.59, ASA: 0.57, Lagarde: 0.60, Padua: 0.80 (0.77-0.83) | HL-test, Pearson goodness of fit | HL: 0.67 | aCCI: Pearson 0.48, CCI: Pearson 0.17, ASA: Pearson 0.60, Lagarde: HL 0.55, Padua: Not measured | aCCI, CCI & ASA: Risk scale, Lagarde: Nomogram, O-Possum & Padua: Equation |
| **Raymond et al, 2016 (50)** | 3 models: 30-day/IHM, morbidity, mortality and/or morbidity | mortality: 135/3942, morbidity: 1429/3942 | N.a. | Mortality: 30-day/IH, morbidity: n.a. | AUC | mortality: 0.71, morbidity: 0.63, mortality and/or morbidity: 0.63 | No validation | HL-test | No data | No data | Equations |
| **Wan et al, 2022 (41)** | 4 models: 30-day morbidity and 30-day mortality | Mortality: 391/10602, morbidity: 4234/10602 | N.a. | 30 days | AUC | N.a. | Morbidity: RAI-rev (cancer corrected) 0.51 (0.50-0.53), RAI-rev 0.54 (0.53-0.56), RAI-A 0.54 (0.53-0.55), mFI-5 0.57 (0.56-0.58). Mortality: RAI-rev (cancer corrected) 0.60 (0.57-0.63), RAI-rev 0.62 (0.59-0.64), RAI-A 0.58 (0.55-0.61), mFI-5 0.58 (0.55-0.61) | No data | N.a. | No data | Risk scales |
| **Saito et al, 2019 (26)** | 1 model: Major morbidity (Clav Dindo≥ 3A) | Major morbidity: 32/90 | N.a. | Unknown | AUC | 0.798 (0.696–0.871) | No validation | No data | N.a. | no data | Risk scale |
| **Scarpa et al, 2016 (30)** | 3 models: 90-day morbidity | Minor complications: 42/181, Major complications: 20/181 | N.a. | 90 days | AUC | N.a. | aCCI: Younger 0.59, elder 0.62, CCI: younger 0.59, elder 0.64, ASA: younger 0.55, elder 0.63 | HL-test | N.a. | aCCI: Younger 0.39, elder 0.40, CCI: younger 0.23, elder 0.56, ASA: Younger 0.73, elder 0.43 | Risk scale |
| **Filip et al, 2014 (28)** | 3 models: 30-day mortality, minor complications (Clav Dindo I or II), major complications (Clav Dindo III-V) | mortality: 5/43, minor complications: 13/43, major complications 14/43 | N.a. | 30 days | AUC | N.a. | aCCI: 0.736 (0.58-0.85), CCI: 0.608 (0.44-0.75), O-POSSUM: N.a. | O/E Ratio | N.a. | O-POSSUM: 1.7, other models not available | aCCI & CCI: Risk scale, O-POSSUM: Equation |
| **Mora et al, 2021 (31)** | 4 models: Morbidity and mortality | Minor morbidity: 96/230, major morbidity: 72/230 | N.a. | Death or LtFU | AUC, sens/spec | N.a. | aCCI: AUC 0.53 (0.44–0.63), spec 0.74, sens 0.90 , CCI: AUC 0.51 (0.42–0.62), spec 0.87, sens 0.97, Steyerberg: AUC 0.56 (0.47–0.66), spec 0.02, sens 0.15, O-Possum: No data | No data | N.a. | No data | aCCI & CCI: Risk scale, O-POSSUM: Equation |
| **Gray et al, 2023 (51)** | 2 models: Different complications | Any complication 94/240, Serious complications 80/240 | N.a. | 30 days | AUC | N.a. | SRC: Any complication: 0.55 (0.48-0.63), Serious complications: 0.55 (0.47-0.63). frailty index: any complication (C-index, 0.51), serious complications (C-index, 0.52). | Calibration plot (alleen SRC) | N.a. | Calibration plot | Equation and risk scale |
| **Ravindran et al, 2020 (52)** | 1 model: Different complications | Serious complication 23/100, Any complication 29/100 | N.a. | 30 days | AUC | N.a. | Serious complication 0.61, Any complication 0.63. | No data | N.a. | No data | Equation |
| ***Anastomotic leakage*** | | | | | | | | | | | |
| **Fischer et al, 2016 (48)** | Dev of 3 models: 30-day mortality, 90-day mortality, anastomotic leakage | 30-day: 112/4882, 90-day: 216/4882 , AL: 305/4882 | N.a. | mortality: 90-day, anastomotic leakage: Unknown | AUC | 30-day: 0.70, 90-day: 0.69, AL: 0.63 | 30-day: 0.646, 90-day: 0.664, AL: 0.587 | Scatterplot | No data | No data | Equations |
| **Van Kooten et al, 2022 (57)** | Dev of 2 models: Anastomotic leakage and pulmonary complications | Anastomotic leakage 799/4228, pulmonary complications 1380/4228 | N.a. | 30 days | AUC | AL: 0.62 (0.58-0.66), pulmonary complications: 0.64 (0.61-0.68) | No data | no data | no data | no data | Nomogram |
| **Ohkura et al, 2019 (22)** | Dev of 8 models: Different complications, among them pneumonia and anastomotic leakage | Pneumonia: 1588/10862, anastomotic leakage: 1371/10862 | Pneumonia 1275/313, anastomotic leakage 1089/282 | 30 days | AUC | No data of development set | anastomotic leakage: 0.53 (0.50-0.57), pneumonia: 0.63 (0.60‐0.67) | No data | no data | no data | Equation |
| ***Pulmonary complications*** | | | | | | | | | | | |
| **Thomas et al, 2019 (33)** | Dev of 1 model: Pulmonary complications (pneumonia, ARDS, resp failure) | 157/691 | 144/13 | 30 days or hospital stay | AUC | 0.71 (0.67-0.73) | 0.79 (0.64–0.88) | Calibration plot | No data | Calibration plot | Equation |
| **Van Kooten et al, 2022 (35)** | Dev of 2 models: Anastomotic leakage and pulmonary complications | Anastomotic leakage 799/4228, pulmonary complications 1380/4228 | N.a. | 30 days | AUC | AL: 0.62 (0.58-0.66), pulmonary complications: 0.64 (0.61-0.68) | No data | no data | no data | no data | Nomogram |
| **Ohkura et al, 2019 (22)** | Dev of 8 models: Different complications, among them pneumonia and anastomotic leakage | Pneumonia: 1588/10862, anastomotic leakage: 1371/10862 | Pneumonia 1275/313, anastomotic leakage 1089/282 | 30 days | AUC | No data of development set | anastomotic leakage: 0.53 (0.50-0.57), pneumonia: 0.63 (0.60‐0.67) | No data | no data | no data | Equation |
| **Kanda et al, 2019 (53)** | Dev of 1 model: Postoperative pneumonia | 41/355 | N.a. | 30 days or hospital stay | AUC | 0.702 | No validation | No data | No data | No validation | Risk scale |
| **Wang et al, 2022 (32)** | Dev of 1 model: Postoperative pneumonia | 26/78 | N.a. | 30 days | AUC | 0.85 (0.75–0.95) | No validation | Calibration slope | 0.98 | No validation | Nomogram |
| **Reinersman et al, 2016 (34)** | Val of 1 model: Major pulmonary complications | Pulmonary complications 47/136 | N.a. | Unknown | AUC | N.a. | Ferguson: 0.76 (0.72-0.81) | HL-test | N.a. | 0.2394 | Risk scale |

**Supplemental Material 6 Validation of models**

Eleven existing models were one or more times validated: Lagarde’s nomogram (1), Steyerberg (2), CCI (4), aCCI (4), ASA score (3), O-POSSUM (4), mFI-5 (2), ACS NSQIP (2), Ferguson (1), RAI-A (1) and RAI-rev (1). Lagarde’s nomogram, Steyerberg’s model, Ferguson’s model and the O-POSSUM model were developed specifically for esophagectomy. The other models were developed for general surgical procedures and in these studies validated for esophagectomy.
